# Accuracy of ultrasound-guided lumbar vertebral level identification: a comparative study with palpation in elderly patients with spinal deformities

**DOI:** 10.1007/s10396-025-01576-7

**Published:** 2025-09-12

**Authors:** Naofumi Hashiguchi, Yasushi Fujiwara, Nanoha Sato, Akiko Matsumoto, Yasushi Murakami, Shinji Kotaka, Ryo Ota, Nobuo Adachi

**Affiliations:** 1 Department of Orthopaedic Surgery, Graduate School of Biomedical and Health Sciences, Hiroshima University, Kasumi, Minami-Ku, Hiroshima City, Hiroshima 734-0037 Japan; 2 Department of Orthopaedic Surgery/ Microscopic Spine and Spinal Cord Center, Hiroshima City North Medical Center Asa Citizens Hospital, 1-2-1 Kameyama Minami, Asakita-Ku, Hiroshima City, Hiroshima 734-0037 Japan

**Keywords:** Ultrasound, Lumbar vertebral identification, Spinal deformities

## Abstract

**Purpose:**

Accurate identification of lumbar vertebral levels is crucial for the success of various interventional procedures, but conventional fluoroscopic guidance exposes both patients and physician to radiation. While ultrasound has emerged as a potential radiation-free alternative, its accuracy in elderly patients with spinal deformities remains unclear.

**Methods:**

In this single-center cross-sectional study, we compared ultrasound-guided versus palpation-guided lumbar level identification in 115 patients scheduled for lumbar surgery between July 2019 and January 2020. Patients were randomly assigned to ultrasound guidance (U group, n = 57) or conventional palpation (P group, n = 58). The primary outcome measure was accuracy of vertebral level identification, verified by intraoperative fluoroscopy.

**Results:**

The U group demonstrated significantly higher accuracy (82.5%) compared to the P group (50.0%) (p = 0.0003, 95% CI [1.5–4.4]). Accuracy was particularly high at the L4 level (U group: 90.3%, P group: 55.9%, p = 0.0023). After adjusting for age and planned needle insertion site, ultrasound guidance maintained superior accuracy (OR = 5.5, 95% CI: 2.3–14.0, p = 0.0002).

**Conclusions:**

Ultrasound guidance provides superior accuracy in lumbar level identification compared to conventional palpation, even in elderly patients with spinal deformities. This technique may offer a reliable, radiation-free alternative, potentially reducing radiation exposure while maintaining high accuracy.

## Introduction

Accurate identification of lumbar vertebral levels is crucial for the success of various interventional procedures, including lumbar facet blocks and nerve root injections [1]. These procedures are commonly used in pain management and require precise localization to ensure efficacy and safety. While fluoroscopic guidance remains the standard method, the cumulative radiation exposure poses significant health risks to both physicians and patients, particularly during complex procedures or repeated interventions [2, 3]. These concerns have led to increased interest in radiation-free alternatives, with ultrasound emerging as a promising solution for preoperative level marking and intraoperative guidance.

Ultrasound guidance offers several distinct advantages beyond radiation avoidance, including dynamic assessment of spinal anatomy and portability [4, 5]. However, previous studies have shown inconsistent results regarding its accuracy [6, 7]. Furness et al. reported 71% accuracy with ultrasound versus 30% with palpation in young adults [8], while Ambulkar et al. found comparable rates between ultrasound (42%) and palpation (48%) in a mixed population [9]. Mieritz et al. demonstrated improved accuracy using standardized ultrasound protocols, suggesting technique refinement could enhance reliability [10]. These inconsistent findings highlight the need for further investigation into factors affecting ultrasound accuracy.

Current evidence has significant limitations for broad clinical application. Most studies have focused on young, healthy subjects with normal spinal anatomy [11, 12], while spine surgeons frequently encounter elderly patients with degenerative changes, deformities, and complex anatomical variations. The accuracy of ultrasound guidance in these challenging cases remains poorly understood. Furthermore, few studies have compared ultrasound accuracy with experienced spine surgeons' palpation techniques, particularly in patients with spinal deformities or a high body mass index (BMI) [13]. These knowledge gaps limit confident implementation of ultrasound guidance in routine clinical practice.

To address these limitations, we designed a retrospective cohort study comparing ultrasound-guided lumbar level identification with the palpation by experienced orthopedic surgeons in a diverse clinical population. Our study specifically included elderly patients and those with spinal deformities to reflect real-world surgical scenarios. We hypothesized that ultrasound guidance would maintain superior accuracy compared to palpation by experienced orthopedic surgeons across different patient subgroups, including those with elderly people or spinal deformities. By evaluating these factors, we aimed to provide practical evidence for implementing ultrasound guidance while potentially reducing radiation exposure in fluoroscopic intervention.

## Materials and methods

### Study design and setting

This single-center cross-sectional study analyzed patients who underwent lumbar spine surgery between July 2019 and January 2020. The first and last authors take complete responsibility for the integrity of the data and the accuracy of the data analysis.

### Participants

**Fig. 1:**
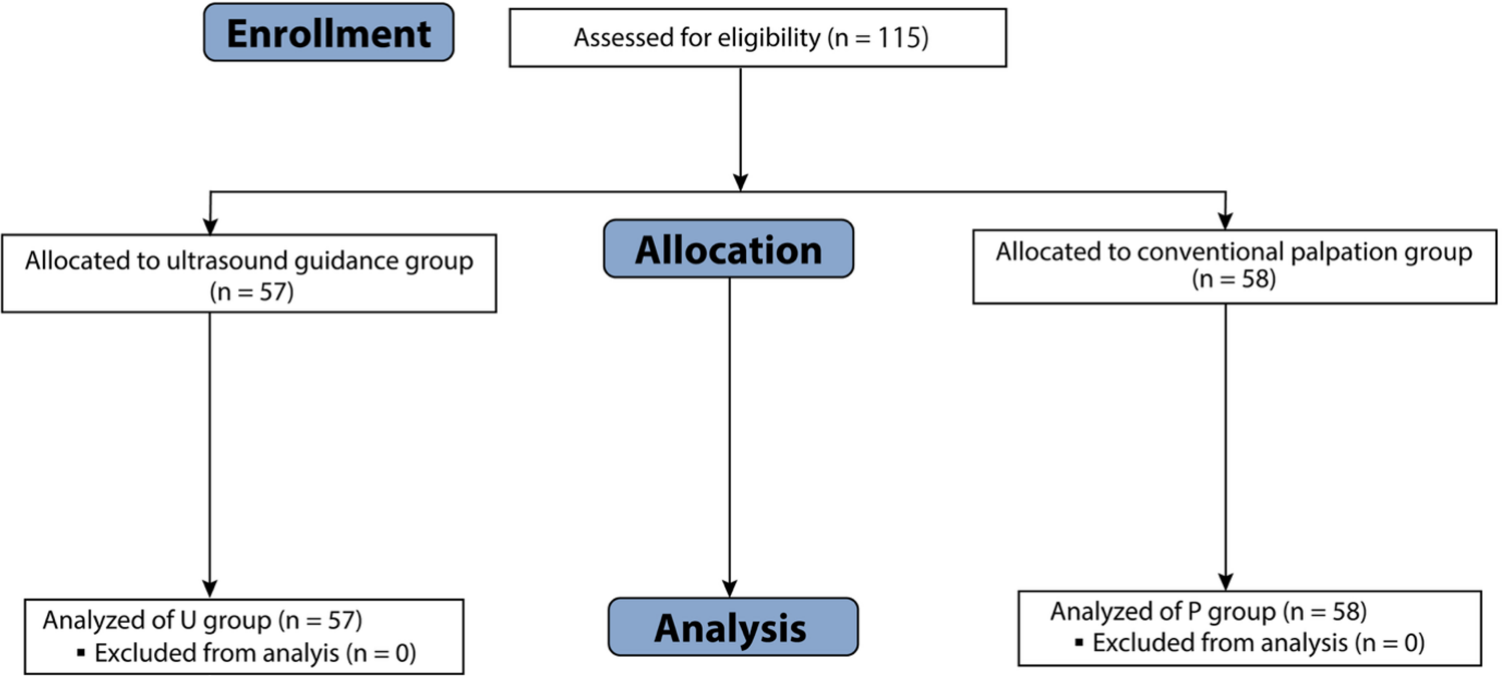
Flow chart of patient allocation

A total of 115 patients (74 males and 41 females) who underwent lumbar spine surgery were included in this study. The mean age of all participants was 66.9 ± 14.7 years (range: 16–91 years). Patients with a history of previous lumbar surgery were excluded to avoid potential confounding factors related to scar tissue and altered anatomy. The study population was randomly assigned in a 1:1 ratio to either ultrasound-guided (U group) or conventional palpation-guided (P group) vertebral level identification. The U group consisted of 57 patients (36 males and 21 females), and the P group comprised 58 patients (38 males and 20 females) (Fig. 1).

### Variables and assessments

Demographic data collected included age, sex, height, body weight, and BMI obtained during preoperative evaluation. Additional variables assessed were the planned insertion site and the presence of spinal deformities at the lumbar level. The spinal deformities evaluated included scoliosis, pre-existing vertebral fractures, spondylolisthesis, facet joint arthropathy, ossification of supraspinous ligament, kissing spine, and spondylolysis.　The presence of spinal deformities was evaluated using preoperative anteroposterior and lateral radiographs. To minimize potential bias, the radiographic assessment was performed by senior residents who were blinded to the study objectives.

### Ultrasound and conventional methods

**Fig. 2:**
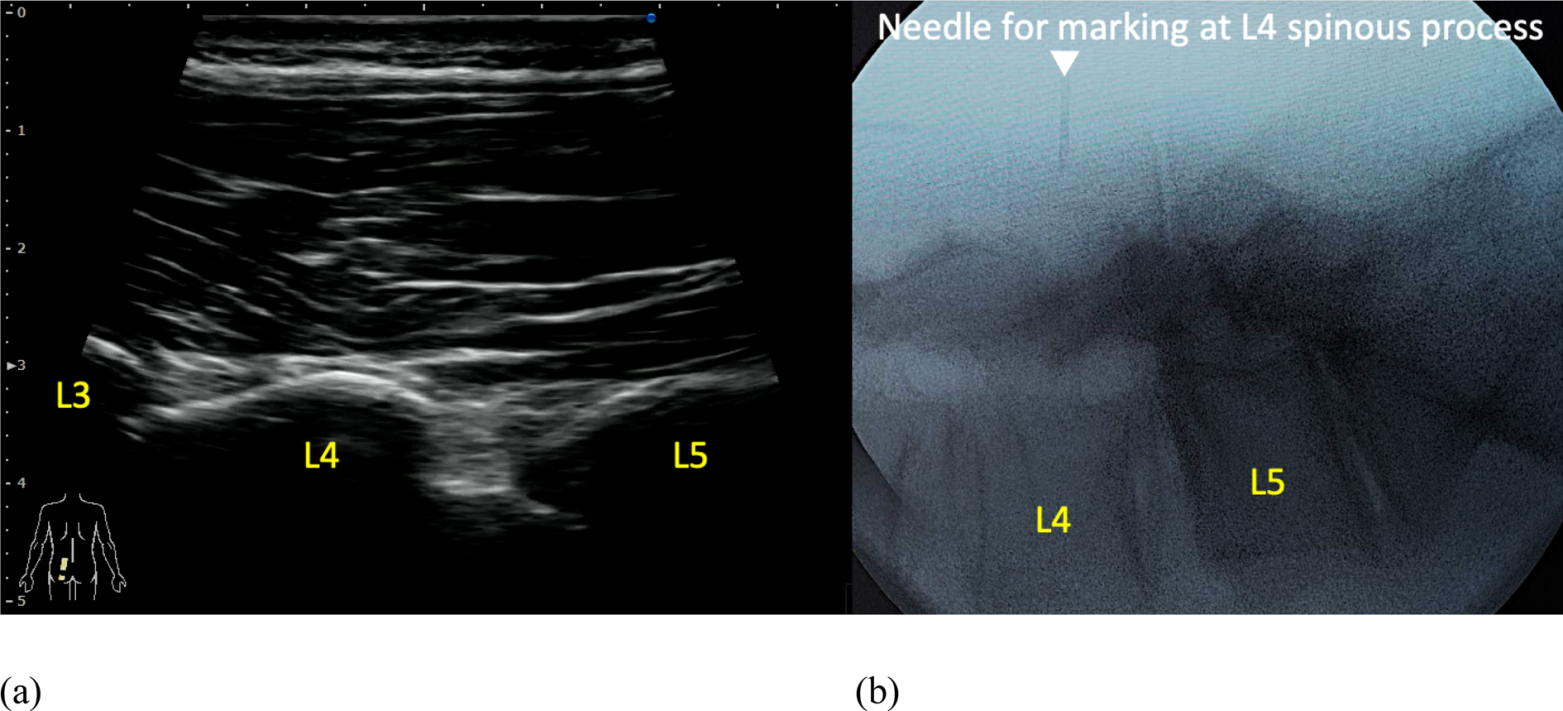
Ultrasound-guided marking of the L4 level with an 18-gauge needle. **a** Paramedian sagittal articular process view, **b** Lateral fluoroscopic imaging

In the ultrasound group (U group), vertebral level identification was performed using the SONIMAGE MX1 ultrasound system (KONICA MINOLTA, Japan) equipped with an L11-3 transducer. All ultrasound examinations were conducted by an experienced orthopedic surgeon (N.H.) with 5 years of clinical ultrasound experience.　Under ultrasound guidance, the target vertebral level was identified from L5/S with the paramedian sagittal articular process view following the previous study [14] and marked by inserting an 18-gauge needle into the spinous process (Fig. 2). The accuracy of level identification was then verified using lateral fluoroscopic imaging. Three spine surgeons, each with more than 15 years of post-graduate experience, evaluated the accuracy of the level identification. In the palpation group (P group), conventional preoperative level identification was performed using manual palpation. One of three spine surgeons, each with more than 15 years of post-graduate experience, identified the target level through palpation and marked it by inserting an 18-gauge needle. The accuracy was similarly verified using lateral fluoroscopic imaging and evaluated by two spine surgeons with more than 15 years of experience. The primary outcome was the comparison of accuracy rates between the ultrasound-guided and palpation-guided vertebral level identification methods and relative risk.　Additionally, we analyzed the accuracy rates for each spinal level compared between the two groups.

### Sample size and statistical analysis

The sample size was calculated based on prior accuracy studies by Hayes et al. (n = 30) [15], Lee et al. (n = 51) [16], and Furness et al. (n = 50) [8], which evaluated lumbar level identification methods. To ensure adequate statistical power (β = 0.2, α = 0.05) for detecting a 30% accuracy difference between groups while accommodating potential anatomical variations in our elderly population with spinal deformities, we allocated ≥ 50 participants per group.

Continuous variables (age, height, body weight, and BMI) were expressed as means with standard deviations. Comparisons of continuous variables between groups were performed using unpaired t-tests. Categorical variables, including sex, planned needle insertion site, and presence of spinal deformities (scoliosis, pre-existing vertebral fractures, spondylolisthesis, facet joint arthropathy, ossification of supraspinous ligament, kissing spine), and accuracy rates of level identification were compared between groups using Fisher's exact test. To evaluate the relationship between accuracy rates and other factors, logistic regression analysis was performed with accuracy rate as the dependent variable and presence of age, ultrasound use, and planned needle insertion site as the independent variables according to the clinical insight. All statistical analyses were performed using GraphPad Prism version 10 (GraphPad Software, San Diego, CA). Statistical significance was set at p < 0.05.

## Results

Demographic data for the U group and P group are shown in Table 1. No significant differences were found in sex, age, height, body weight, or BMI between groups. Table 2 shows the planned puncture sites and presence of spinal deformities, with no significant differences between groups. The accuracy of vertebral level identification was significantly higher in the U group (82.5%, 47/57 cases) compared to the P group (50.0%, 29/58 cases), with a relative risk of 2.4 (p = 0.0003, 95% CI [1.5–4.4]) and the stratified analysis for each vertebral level yielded the results shown in Table 3.

**Table 1:** Demographic Data of Patients*

	U group (N = 57)	P group (N = 58)	P Value^十^
Age (yr), mean ± SD	65.8 ± 15.0	68.0 ± 14.5	0.43
Female sex (n [%])	24 (42.1%)	20 (34.5%)	0.45
Height (m)_,_ mean ± SD	1.62 ± 0.1	1.60 ± 0.1	0.51
Weight (kg), mean ± SD	64.1 ± 14.0	62.8 ± 12.2	0.58
BMI (kg/m/m), mean ± SD	24.5 ± 4.2	24.4 ± 3.8	0.90
Planned needle insertion site			
L1 (n [%])	0 (0%)	1 (1.7%)	
L2 (n [%])	3 (5.3%)	8 (13.8%)	
L3 (n [%])	18 (31.6%)	6 (10.3%)	
L4 (n [%])	31 (54.4%)	35 (60.3%)	
L5 (n [%])	5 (8.8%)	8 (13.8%)	

*The values are given as the mean ± standard deviation (SD) for continuous variables and the number and percentage (%) for categorical variables. U group, ultrasound-guided group; P group, palpation-guided group.

十 Unpaired t test (age, height, body weight, and BMI) and Fisher's exact test (sex and planned needle insertion site) for between-group comparisons

**Table 2:** Spinal deformities at the lumbar level*

	U group (N = 57)	P group (N = 58)	P Value^十^
Scoliosis (n [%])	12 (78.9%)	19 (67.2%)	0.21
Vertebral fractures (n [%])	9 (15.8%)	11 (19.0%)	0.81
Spondylolisthesis (n [%])	16 (28.1%)	24 (41.4%)	0.17
Facet joint arthropathy (n [%])	49 (86.0%)	55 (94.8%)	0.12
Ossification of supraspinous ligament,(no.[%])	13 (22.8%)	19 (32.8%)	0.30
Kissing spine (n [%)	38 (66.7%)	38 (65.5%)	> 0.99
Spondylolysis (n [%])	2 (3.5%)	4 (6.9%)	0.68

*The values are given as the number (n) and percentage (%) for categorical variables. U group, ultrasound-guided group; P group, palpation-guided group.

十Fisher's exact test for between-group comparisons

**Table 3:** Accuracy Comparison

	Accuracy Comparison*		P Value^十^
	U group (N = 57)	P group (N = 58)	
ALL (n [%])	47/57 (82.5%)	29/58 (50.0%)	**0.0003**
L2 (n [%])	1/3 (33.3%)	2/6 (25.0%)	> 0.99
L3 (n [%])	15/18 (83.3%)	3/6 (50.0%)	0.14
L4 (n [%])	28/31 (90.3%)	19/34 (55.9%)	**0.0023**
L5 (n [%])	3/5 (60.0%)	4/8 (50.0%)	> 0.99

*The values are given as percentage (%) for accuracy rate. U group, ultrasound-guided group; P group, palpation-guided group. 十 Fisher's exact test for between-group comparisons

**Fig. 3:**
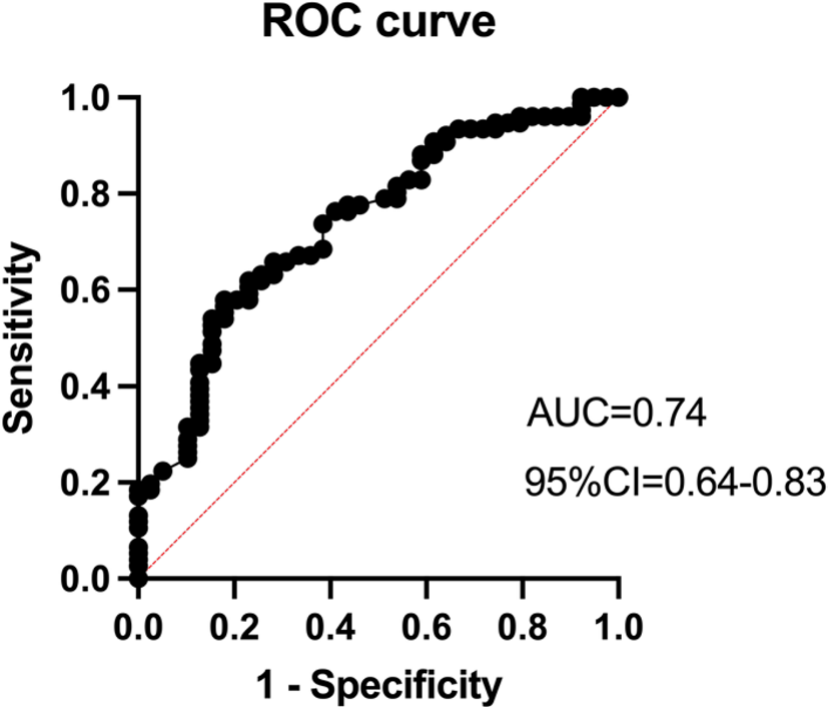
ROC curve

The logistic regression model showed good discrimination (Fig. 3). The overall accuracy rate was 73.0%, with a sensitivity of 90.8% and specificity of 38.5%. Ultrasound use and planned needle insertion site significantly predicted accuracy. The U group was associated with higher accuracy compared to the P group (OR = 5.5, 95% CI: 2.3–14.0, p = 0.0002). The lower lumbar needle insertion position (L4) predicted better accuracy (OR = 1.7, 95% CI: 1.0–3,0, p = 0.04). Age did not significantly affect accuracy (OR = 1.0, 95% CI: 0.9–1.1, p = 0.1). The model showed a positive predictive value of 74.2% and a negative predictive value of 68.2%. These results suggest that US guidance provides better accuracy than palpation, and accuracy improves with lower lumbar needle insertion position (L4).

## Discussion

In this study, we found that ultrasound-guided vertebral level identification demonstrated significantly higher accuracy than traditional palpation methods. The ultrasound group achieved an accuracy rate of 82.5%, while the palpation group showed an accuracy rate of 50.0% (p = 0.0003, 95% CI [1.45–4.35]). The accuracy was particularly notable at the L4 level, where the ultrasound group achieved a 90.3% success rate compared to 55.9% in the palpation group (p = 0.0023, 95% CI [1.45–10.42]). After adjusting for age and planned needle insertion site, multivariate analysis revealed that ultrasound guidance was associated with significantly higher accuracy compared to palpation (OR = 5.56, 95% CI: 2.27–14.08, p = 0.0002). These findings clearly demonstrate that ultrasound guidance provides superior accuracy in lumbar level identification compared to traditional palpation methods, particularly at the L4 level, which is crucial in clinical practice.

Recent years have seen an increase in ultrasound-guided facet joint and nerve root blocks in the lumbar region, making accurate vertebral level identification increasingly critical [17]. Previous studies have reported wide variations in accuracy rates for vertebral level identification, with ultrasound guidance showing 34–71% accuracy and palpation methods ranging from 12 to 69% [8, 9, 16, 18, 19]. Additionally, other studies encompassed diverse target populations, including children [20], pregnant women [17, 21–24], and adults aged 40–50 years [8, 13, 19]. Animal studies have also confirmed the effectiveness of ultrasound, while in humans, various factors may be involved [25].

Several factors influence identification accuracy, including examiner experience, anatomical variations, patient characteristics such as obesity and sex, and abdominal circumference [26]. While previous studies have shown that accuracy typically decreases with age [15], particularly in elderly patients and infants, our study achieved an 82.5% accuracy rate with ultrasound guidance despite including many elderly patients with spinal deformities, similar to a previous prospective randomized trial [27]. Notably, ultrasound guidance maintained significantly higher accuracy compared to palpation even after adjusting for anatomical variations, obesity, sex, and age, representing a possibility of a novel finding in this field.

Previous studies have shown varying accuracy rates for ultrasound-guided vertebral level identification across different lumbar levels. Patil et al. studied 50 patients (mean age 46.7 years) with lumbosacral degenerative diseases and found that identification accuracy was highest at L4-L5, moderate at L5-S1, and lowest at L3-L4, with statistically significant differences between levels (p = 0.016) [13]. Furness et al. examined 50 patients (mean age 45 years) with lumbar radiographs and reported identification accuracy rates of 60% at L2-L3 and 71% at both L3-L4 and L4-L5 levels, with no cases at L5-S1 [8]. Similarly, Lee et al. studied 51 late-term pregnant women (mean age 29 years) and reported comparable accuracy rates across different levels [16].

In contrast, previous studies did not specify which scanning plane was used [8, 24]. Our methods section, however, clearly defines the paramedian sagittal articular process view. An obstetric neuraxial block study using a paramedian sagittal approach reported 68–76% accuracy rising to 90% after training [28]. By using the paramedian sagittal articular process view, we achieved 90.3% accuracy at L4. This confirms that selecting the paramedian view is key to improving vertebral level identification accuracy.

Our findings align with these previous studies, demonstrating high accuracy (90.3%) at the L4-L5 level, despite our older study population (mean age 66.9 years). The high accuracy at L4-L5 can be attributed to the systematic identification method, where L4 is located by first identifying the L5 lamina and then tracking upward. However, accuracy may tend to decrease for higher lumbar levels as the distance from the initial reference point increases [14]. Additionally, there is a potential risk of misidentifying L5 as S1, which highlights the importance of careful anatomical assessment during the procedure.

Several limitations should be noted in this study. First, while we assessed the presence of spinal deformities such as scoliosis, we did not evaluate their severity or degree of deformation. The relationship between deformity severity and accuracy of vertebral level identification remains unclear and warrants further investigation. Second, our study suggests that ultrasound accuracy may decrease when identifying upper lumbar levels, possibly due to increased depth and anatomical complexity. However, due to the limited sample size for upper lumbar vertebrae, we could not perform a comprehensive statistical analysis of level-specific accuracy. Future studies with larger cohorts are needed to validate these findings and establish level-specific reliability.

However, the novelty of this study lies in its evaluation of ultrasound accuracy in a clinically relevant population of elderly patients with spinal deformities. While previous studies have mainly focused on younger patients (40–50 years olds) [8, 13, 19] or those with normal spinal anatomy, our study provides practical evidence for ultrasound guidance in a challenging patient population that spine surgeons frequently encounter. Our findings demonstrate that ultrasound maintains superior accuracy compared to palpation even in the presence of age-related anatomical changes and spinal deformities.

While our results suggest that the accuracy achieved may not yet meet the precision required for lumbar surgery level marking, our primary aim was to assess ultrasound guidance as a noninvasive tool for pre-procedure planning in outpatient settings such as facet joint blocks and nerve root injections. Our findings support its use as a reliable aid for these interventional procedures, with particularly high accuracy at the L4 level (90.3%) being especially beneficial.

Future research should focus on further improving ultrasound guidance accuracy and evaluating its clinical efficacy in various interventional procedures. These efforts will enhance the role of ultrasound in spine care while potentially reducing radiation exposure for both patients and healthcare providers.

## Conclusions

This study demonstrates that ultrasound offers significantly higher accuracy in lumbar vertebral level identification compared to traditional palpation methods, achieving an accuracy rate of 82.5% versus 50.0%. This superior performance was maintained even in elderly patients with spinal deformities, highlighting its potential as a reliable tool for pain management procedures such as lumbar facet blocks and nerve root injections. By providing a radiation-free alternative, ultrasound guidance may enhance precision while reducing the risks associated with fluoroscopic exposure.

## Data Availability

The datasets generated and analyzed during the current study are not publicly available due to ethical and privacy restrictions; however, they are available from the corresponding author upon reasonable request.
